# The relationship between fathers' heavy episodic drinking and fathering involvement in five Asia‐Pacific countries: An individual participant data meta‐analysis

**DOI:** 10.1111/acer.14955

**Published:** 2022-12-16

**Authors:** Anne‐Marie Laslett, Sandra Kuntsche, Ingrid M. Wilson, Angela Taft, Emma Fulu, Rachel Jewkes, Kathryn Graham

**Affiliations:** ^1^ Centre for Alcohol Policy Research La Trobe University Melbourne Victoria Australia; ^2^ National Drug Research Institute Curtin University Perth Western Australia Australia; ^3^ Melbourne School of Global and Population Health University of Melbourne Melbourne Victoria Australia; ^4^ Singapore Institute of Technology Singapore Singapore; ^5^ Judith Lumley Centre La Trobe University Melbourne Victoria Australia; ^6^ Department of Sociology, Social Policy and Criminology University of Liverpool in Singapore Singapore Singapore; ^7^ The Equality Institute Melbourne Victoria Australia; ^8^ Office of the Executive Scientist South African Medical Research Council (SAMRC) Pretoria South Africa; ^9^ Centre for Addiction and Mental Health Toronto/London Ontario Canada; ^10^ Dalla Lana School of Public Health University of Toronto Toronto Ontario Canada

**Keywords:** child trauma, fathering involvement, men's heavy episodic drinking

## Abstract

**Background:**

This study aims to increase understanding of the relationship between heavy episodic drinking (HED) and fathers' involvement in parenting in five countries. The potential moderating effect of fathers' experiences of childhood trauma is also studied, controlling for the possible confounding of the effect of HED by father's attitudes toward gender equality, father's age and father's education.

**Method:**

United Nations Multi‐Country Study on Men and Violence (UNMCS) survey data from 4562 fathers aged 18–49 years from Cambodia, China, Indonesia and Papua New Guinea (PNG) and Sri Lanka were used to assess the relationship between fathering involvement (e.g., helping children with their homework) and self‐reported HED of 6+ drinks in one occasion vs. non‐HED and abstaining. Moderating effects of a 13‐item fathers' childhood trauma (FCT) scale were tested and analyses were adjusted for gender‐inequitable attitudes using the Gender‐Equitable Men scale score. Bivariate and adjusted individual participant meta‐analyses were used to determine effect estimates for each site and across all sites.

**Results:**

Fathers' HED was associated with less positive parental involvement after adjusting for gender‐equitable attitudes, FCT, age and education. No overall interaction between HED and FCT was identified. Gender equitable attitudes were associated with fathering involvement in some countries but not overall (*p* = 0.07).

**Conclusions:**

Heavy episodic drinking was associated with reduced positive fathering involvement. These findings suggest that interventions to increase fathers' involvement in parenting should include targeting reductions in fathers' HED. Structural barriers to fathers' involvement should be considered alongside HED in future studies of fathers' engagement with their children.

## Graphical abstract

In the current study, we fed mice for 8‐weeks with chronic ethanol plus one or three binges and performed microarray on liver samples. Liver cytochrome P450 2B10 (CYP2B10) expression and protein levels were highly induced by binge ethanol. We generated CYP2B10‐knockout mice and found that they exhibit increased levels of liver injury after chronic‐plus‐binge ethanol administration. CYP2B10 plays a protective role in ethanol‐induced liver injury by indirectly increasing ethanol metabolism and altering liver metabolites.

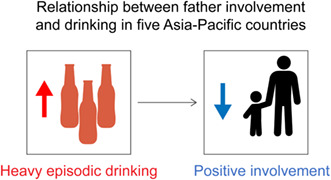

## BACKGROUND

Fathers around the world are increasingly involved in children's development (Gerson, 2009; van der Gaag et al., 2019). Fathering and parenting generally can include being emotionally available for children, doing things with children, including playing with them and reading to them, as well as providing for and teaching them (Gerson, 2009; Mehus et al., 2021). There are many facets to fathering and fathers can, of course, have negative and positive influences on their children as they engage with them. The overarching study and variable selection was informed by WHO's model for understanding violence against women (Fulu, Warner, et al., 2013; Heise, 1998). However, this paper also draws on elements of models of understanding child development (Bronfenbrenner, 1979), Fox and Bruce's use of Identity Theory and Parental Investment Theory (Fox & Bruce, 2001), understanding of masculinities and fatherhood (Schoppe‐Sullivan et al., 2021), masculinities and drinking (de Visser & McDonnell, 2012; Iwamoto et al., 2011; Moore et al., 2017; Wells et al., 2014) and basic public health socio‐ecological models that view alcohol as a risk factor for problematic behaviors and poorer health and social outcomes (WHO, 2002).

Studies have focused on the role of problematic alcohol and its impact on intimate and family relationships, children's emotional development and subsequent alcohol use (Laslett et al., 2015; Laslett & Cook, 2019). The extent that fathers drink heavily outside the home and around children in the home varies within and between countries (FARE, 2013; Laslett, Stanesby, et al., 2020; Mehus et al., 2021). In Indonesia, 11% of men reported heavy episodic drinking (HED; Laslett, Graham, et al., 2021), while 53% of fathers in Chile reported doing so (Laslett, Stanesby, et al., 2020). Heavy drinking is linked to child abuse and neglect (Laslett et al., 2013). Drinking also likely impacts upon fathers' relationships with their children, although exact effects are not clear. Research directly examining whether men's drinking is associated with reduced engagement with their children is limited. Studies have described how some fathers when drinking or hungover are less available emotionally for their children, potentially more disinhibited around their children, report frustration and stress and may become angry and violent (Freisthler et al., 2020; Laslett, Stanesby, et al., 2020; Lee et al., 2011; Tamutienė & Laslett, 2016; Wilson et al., 2017).

However, less is known about how paternal alcohol use – specifically HED – affects parenting. Attitudes that support traditional gender roles are expected to be associated with less involvement in parenting by men (Bulanda, 2004; McGill, 2014). The present study explores how HED is related to fathers' involvement in parenting. We focus on the relationship between HED and fathering among men in five countries, taking into account other factors that have been shown to be associated with men's actions within families, including fathers' experiences of childhood abuse and gendered attitudes.

How men act as fathers may be impacted by their own experiences as children (Belsky et al., 1984). Fathers' own adverse childhood experiences – including exposure to parental harmful alcohol use – affect their own development, their mental health, their own use of substances and consequently their own intimate relationships, family functioning, their children's development and crucially their relationships with their children (Choenni et al., 2017; English et al., 2015; Schilling et al., 2007; Staton‐Tindall et al., 2013). Thus, men's experience of childhood trauma may potentiate any relationship between HED and involvement in parenting. Hence, in this study, we test for moderation and not simple confounding of this relationship.

Patriarchal systems and economic drivers also operate and support men's behaviors within families (Lesch & Adams, 2016). The ways in which men and fathers act, parent and drink are inseparably linked to understandings of masculinity, roles and many other complex factors (McGill, 2014; Moore et al., 2017) in different settings (MacLean et al., 2020). Schoppe‐Sullivan et al. (2021) found that men who reported more strongly identifying with their role as a father and who reported spending more time with their children, reported higher quality of relationships with their children. Additionally, research in the US has found more equitable gendered attitudes and positive constructions of masculinity to be associated with greater parenting involvement by men when measured by fathers' time spent in leisure activities, playing with their children, helping them with their homework or reading, talking with them in private and watching television or videos together (Bulanda, 2004; McGill, 2014) and when measured as time spent caring for children (Baxter, 2015; Jacobs & Kelley, 2006). Moreover directly, drinking is integrally linked to formations of masculinity and that “conforming to masculine norms or the beliefs and expectations of what it means to be a man, may explain patterns of problematic drinking among men.” (p. 906, Iwamoto et al., 2011). Masculine norms have been associated with HED and drinking consequences, both directly and indirectly via HED (Wells et al., 2014).

Thus, although childhood trauma, gender‐equitable attitudes and alcohol consumption have been linked to parenting, they have not been examined within the same analyses to study positive involvement in fathering. Using the same data as used in the present analyses, Fulu et al. (2017) found that men's experience of childhood abuse was associated with their own harsh parenting practices, that alcohol was associated with perpetration of intimate partner violence (IPV), and that perpetration of IPV led to harsh parenting, but did not examine the direct relationship between alcohol and involvement in parenting.

In this study of fathers in five low‐ and middle‐income countries (LMIC) in the Asia Pacific region, we test the key hypothesis that fathers' current HED is associated with less involvement in parenting, taking into account the possible moderating effect of fathers' childhood trauma (FCT) and controlling for gender inequitable attitudes, as well as men's age and education.

## METHODS

### Data

The present study uses the individual‐level data from the United Nations Multi‐Country Study on Men and Violence (UNMCS) data (Fulu, Warner, et al., 2013). Access and ethical approval were provided by the Sexual Violence Research Initiative (hosted by the South African Medical Research Council for the UNMCS). Additional Ethical approvals were obtained from local institutions or national ethics boards in each country and from La Trobe University for the secondary data analysis (HEC19241, 4 July 2019). All men in the study were aged 18–49 years. Each sample was randomly drawn within selected representative areas of each country. The present analysis includes men with children from 14 sites in five countries: Cambodia (five sites), China (one combined rural/urban site), Indonesia (rural – Purworejo and West Papua and urban ‐ Jakarta), Papua New Guinea (Bougainville) and Sri Lanka (rural, urban sites). These sites are described in detail by Fulu, Jewkes, et al. (2013). Some sites asked not to be named to protect site and participant confidentiality while others requested that they be identified to differentiate between sites with different attributes. Within these regions, neighborhoods, villages, census units or electoral areas were selected using probability proportional to size (PPS), except in China where individuals were sampled from neighborhoods and villages using the district population register. The individual response rates within each country varied from 58.7% to 92.7% (Fulu, Warner, et al., 2013).

### Outcome measures

Father involvement with children (fathering involvement) was assessed with three items measured on a 4‐point Likert scale (never, sometimes, often, and very often), asking the participant about time spent: playing with or participating in activities with his children; discussing personal matters with his children; and helping children with their homework (range: 3–12; Chan et al., 2017). The items ask about the frequency of current parenting practices and do not specify whether these practices were undertaken in the previous year.

### Independent variables

#### Heavy episodic drinking

Alcohol consumption was measured using two questions from the AUDIT screen (Allen et al., 1997; Saunders et al., 1993). Respondents who answered “never” in response to the first question “How often do you drink alcohol?”, were classified as abstainers. The remainder were asked “How often do you have six or more drinks on the one occasion?” (response options: never, less than monthly, monthly, weekly, daily or almost daily): non‐HED was reported for those who reported drinking, but never six or more. For regression analyses respondents were dichotomised into those who reported ever drinking six or more drinks on one occasion (1) vs. non‐HED drinking and abstaining (0).

#### Fathers' childhood trauma

The childhood trauma scale developed by Bernstein et al. (1994) was adapted for the UNMCS (Fulu, Warner, et al., 2013; Jewkes et al., 2012) and measures 13 experiences of past abuse reported by men when they were a child under the age of 18, with the scale including physical, sexual and emotional abuse and emotional and physical neglect. Scores range between 13 and 52, with higher scores indicating greater trauma (Fulu et al., 2017).

#### Gender‐equitable attitudes

The Gender‐Equitable Men (GEM) Scale (Pulerwitz & Barker, 2008) has been used widely and tested in LMIC (Fleming et al., 2015; Gottert et al., 2016) to measure men's attitudes toward gender norms related to women's role in the home, sexual and reproductive health, sexual relations, violence, domestic work and homophobia. Ten items scored on a 4‐point scale from strongly agree to strongly disagree were summed to create a continuous score ranging from 10–40. Higher scores indicate more gender‐equitable attitudes (Singh et al., 2013).

#### Father's age

Father's age was categorized in three categories (18–24, 25–34 and 35–49 years). The youngest age group only contained 180 fathers and was combined, creating two age groups: 18–34 years (1) and 35–49 years (0).

#### Education level

Finally, it is important to control for social status (as measured by education level; Grittner et al., 2012) which is often associated with a variety of health and social outcomes. Educational level was categorized as no high school education (1) versus some or completed high school education (0) and included as a control variable in the adjusted multivariable meta‐analysis final model.

### Analyses

Descriptive, bivariate and adjusted individual participant meta‐analyses that took account of country‐level stratification and site‐level clustering and individual participant data were used to produce prevalence estimates and effect estimates between specified variables for the combined sample. We used individual participant [or patient] data meta‐analysis (ipdmetan), which analyses the individual, site, and country level data, and pools the data from all sites. The ipdmetan adjusts for individual level and group level effects, thereby in the analysis acting as a form of multi‐level analysis, adjusting for both individual and country level effects. The random effects meta‐analysis allows for variability across sites and countries while increasing power through pooled sample size. Analyzing individual sites or countries may result in underpowered analyses, while the meta‐analytic approach increases power to detect an effect, and the random effects account for some of the site‐specific and country‐specific sources of variation. Ipdmetan is a useful tool for adjusting for multiple studies while allowing analysis of multiple surveys gathered using the same survey tools, where the individual data are available for secondary data analysis. Random effects individual‐level participant meta‐analysis was employed because, while the survey items were identical, the studies varied by cultural background and sample composition (Borenstein et al., 2007; Huedo‐Medina et al., 2006). Individual participant data meta‐analysis results in an overall mean estimate pooled across all sites and it accounts for variability that captures the error of the estimate plus systematic differences across sites. Country‐level percentages of the populations affected and 95% confidence intervals were calculated, with significant country‐level differences defined as non‐overlapping confidence intervals (du Prel et al., 2009; Gardner & Altmann, 1986). The *I* ^2^ statistic indicates variability in effect sizes due to heterogeneity across studies. *I* ^2^ values of 25%, 50%, and 75% indicate low, medium, and high heterogeneity (Fisher, 2015). We used the DerSimonian‐Laird method of two‐stage inverse‐variance random‐effects meta‐analysis (via the ipdmetan command) using Stata 14.0 (Fisher, 2015) to estimate the pooled effect linear regression estimates (regression co‐efficients) of the dependent variable (fathering involvement) being associated with independent variables (e.g., FCT, HED and GEM‐scores) and pooled interaction effects. The forest plots, in their final form, include the outcome and the key independent variable, adjusting for the specified variables where specified, e.g., the effect of less fathering involvement associated with HED, including the effects of lower GEM scores and FCT, adjusting for individual participant, study site and country results. The random effects meta‐analysis applies weights to each study estimate when pooling, and these weights account for both within‐ and between‐study variance, meaning that it is possible to generalize across countries and estimate pooled effects, particularly when covariates in the model are adjusted for simultaneously. Missing data were relatively equally distributed across variables and sites and comprised less than 5% of variables in the models overall. Missing data were deleted listwise. In the final multivariate ipdmetan model, we also adjusted for father's education and father's age.

## RESULTS

Table 1 presents descriptive data on alcohol consumption, childhood trauma, gender‐equitable attitudes and father involvement for each country. Rates of HED were highest in PNG and Cambodia, in between in China and Sri Lanka, and lowest in Indonesia. Indeed, the majority of men from Indonesia reported abstaining from alcohol, while 24.1–35.4% of men in the other countries reported abstaining. Among drinkers, the distributions of participants who reported drinking six or more drinks on the one occasion never, less than monthly, monthly, weekly, daily or almost daily also indicate that the majority of HED in each country drank less than monthly or monthly. The mean scores for the unadjusted GEM scales reported (i.e., gender‐equitable attitudes) were lower in PNG and Cambodia.

**TABLE 1:** Sample characteristics by country including fathers' alcohol use, childhood trauma, gender‐equitable attitudes and fathering involvement

	Cambodia	China	Indonesia	PNG	Sri Lanka	Total
Survey, *n*	1812	998	2576	864	1533	7783
Fathers in sample, *n*	1149	718	1557	525	613	4562
Alcohol use, % (CI)						
Abstainer	29.5 (26.9, 32.2)	36.2 (32.8, 39.8)	78.0 (75.8, 80.0)	24.2 (20.7, 28.0)	35.4 (31.7, 39.3)	57.9 (56.6, 59.2)
Drinks but never ≥6 on the one occasion	26.2 (23.7, 28.8)	31.6 (28.3, 35.1)	7.6, (6.4, 9.0)	12.4 (9.8, 15.5)	35.9 (32.2, 39.8)	16.6 (15.7, 17.6)
Drinks and has had 6 or more on the one occasion: HED, %(CI)	44.3 (41.5, 47.2)	32.2 (28.9, 35.7)	14.5 (12.8, 16.3)	63.4 (59.2, 67.5)	28.7 (25.3, 32.4)	25.5 (24.4, 26.6)
Frequency of HED (*n* = 2405)						
%never	37.2 (33.6, 40.9)	49.6 (45.0, 54.2)	34.4 (28.3, 41.0)	16.3 (13.0, 20.4)	55.6 (50.3, 60.7)	38.7 (36.5, 40.9)
% < monthly	44.2 (40.5, 48.0)	32.5 (28.9, 36.4)	27.7 (24.3, 31.4)	43.7 (38.7, 48.9)	22.7 (18.1, 28.2)	36.0 (34.1, 38.0)
% monthly	6.8 (5.0, 9.2)	10.7 (8.5, 13.4)	22.5 (18.3, 27.3)	24.4 (20.8, 28.4)	12.6 (9.4, 16.8)	13.6 (12.3, 15.1)
% weekly	6.8 (5.4, 8.5)	4.8 (3.1, 7.4)	11.7 (8.6, 15.6)	11.8 (9.3, 14.9)	6.1 (3.5, 10.4)	7.8 (6.8, 9.0)
% daily or almost daily	5.1 (3.8, 6.7)	2.4 (1.2, 4.7)	3.8 (2.0, 7.1)	3.8 (2.4, 5.8)	3.0 (1.4, 6.6)	3.8 (3.1, 4.7)
Fathers' childhood trauma score, mean (SD)	17.4 (3.1)	16.4 (3.1)	15.9 (2.8)	19.4 (3.9)	16.8 (3.4)	16.9 (3.3)
GEMS score, mean (SD)	22.0 (4.3)	27.9 (3.7)	23.4 (2.8)	22.7 (4.4)	24.9 (5.5)	23.8 (4.4)
Father involvement with children score, mean (SD)	6.4 (1.8)	6.8 (1.4)	7.0 (1.7)	7.2 (1.8)	6.1 (1.8)	6.6 (1.7)

*Note*: The Gender‐Equitable Men (GEM) scale is reported in this table in its original form to be comparable with other papers (The remainder of the paper describes and discusses gender‐inequitable attitudes).

The involvement in fathering scores ranged between 3 and 12, and the mean scores for fathers' involvement varied from 6.1 in Sri Lanka to 7.3 in PNG. The individual components of the score are described in detail for the study elsewhere (Chan et al., 2017), but in this sample, 6.3% reported never, 43.1% reported sometimes, 40.3% reported often and 10.2% reported very often playing or undertaking activities with their children. The respective percentages for talking about personal matters with their children were: 34.1% never; 42.1% sometimes; 20.6% often; and 3.2% very often. Regarding whether fathers reported helping children with their homework: 25.0% reported never; 40.8% sometimes; 28.2% often and 6.0% reported very often doing so.

### Heavy episodic drinking and fathering involvement

**FIGURE 1:**
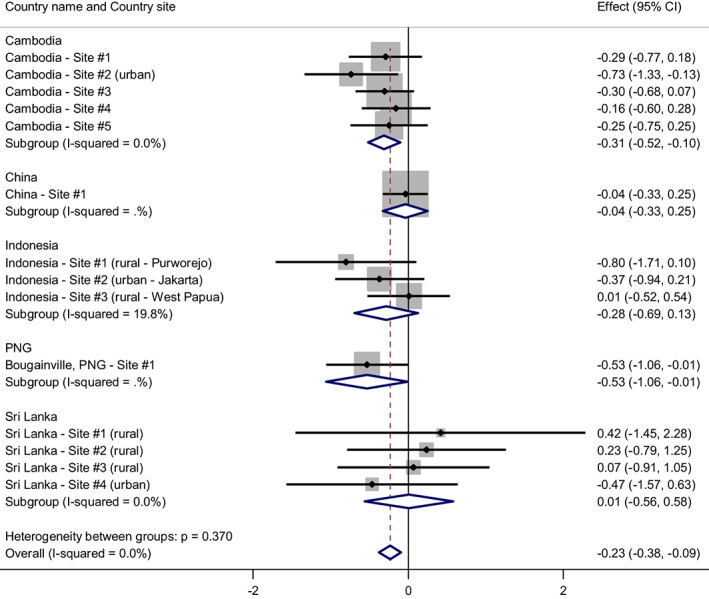
The relationship between heavy episodic drinking and fathering involvement. *Note*: Weights are from random‐effects model

Interpreting Figure 1, HED was associated with less fathering involvement. This relationship was significant in Cambodia, PNG and overall, with an overall difference score of −0.23 (points) when HED was compared to non‐HED drinkers and abstainers. The effect was not significant within three of the five countries and near zero in China and Sri Lanka. This suggests that HED did not influence father involvement in China or Sri Lanka (although the Chinese cohort by itself was somewhat underpowered). We have pooled results and “trials” – here countries – to gain increased capacity to detect overall significant differences and found evidence of a weak effect across all countries. Although not significant, the relationship was also in the predicted direction (HED associated with less fathering involvement) in Indonesia.

### Potential moderation of fathers' childhood trauma on the relationship between HED and fathering

We analyzed the interaction of FCT and HED in predicting fathering involvement to assess whether FCT moderated the relationship of HED with fathering involvement (Figure S1a shows univariate relationship and S1b presents the interaction). No evidence of a significant interaction was found overall, although there was a significant interaction in PNG. Univariately, in PNG, HED was associated with less fathering involvement and FCT was not associated with fathering involvement, but when both were included neither were significant, yet the interaction term was. This indicates that the relationship between father's HED and fathering involvement, was in part moderated or driven by father's experience of trauma as a child. Fathers in PNG who had experienced greater child trauma were more likely to report drinking in a HED way and in turn less likely to be involved with their children.

### Relationship between gender‐inequitable attitudes and fathering

**FIGURE 2:**
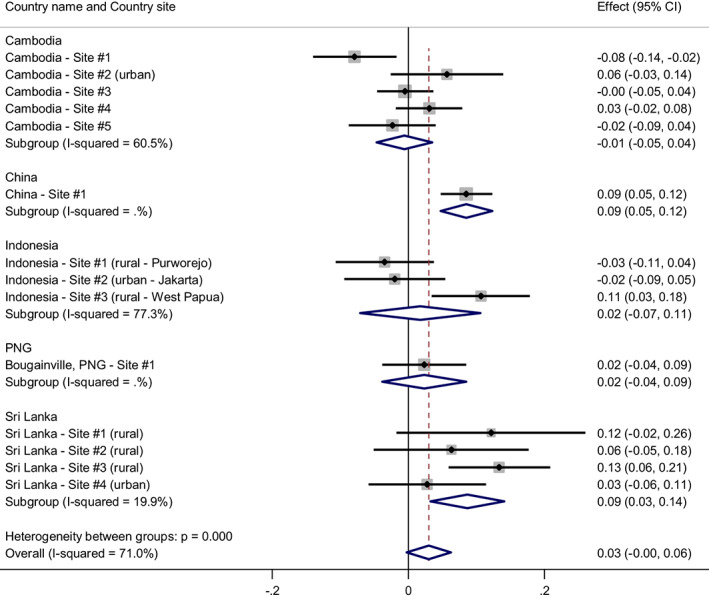
The relationship between higher gender‐equitable attitude scores and fathering involvement. *Note*: Weights are from random‐effects model

Analysis of the relationship between scores on gender‐equitable attitudes and fathering involvement found more gender equitable attitudes were generally linked to more involved parenting (see Figure 2), significantly for China and Sri Lanka, and approaching significance for the analysis across countries (*p* = 0.071); however, there was considerable variability among countries and sites within countries.

#### Analysis of other univariate effects

Younger fathers (18–34 years) reported significantly less involvement with their children than older fathers (35–49 years) overall (effect: −0.35, CI: −0.54, −0.15; *p* < 0.001), and in Cambodia (effect: −0.32, CI: −0.53, −0.12, *p* = 0.002), Indonesia (effect: −0.68, CI: −0.92, −0.45, *p* < 0.0001) and PNG (effect: −0.57, CI: −1.10, −0.05, *p* = 0.032). Non‐significant effects were found for China (effect: 0.17, CI: −0.12, 0.47) and Sri Lanka (effect: −0.10, CI: −0.59, 0.40). Father's education was also significant with any high school education associated with increased engagement with children overall (effect: 0.38, CI:0.23, 0.53) and in Cambodia (effect: 0.44, CI:0.22, 0.65) and Indonesia (effect: 0.30, CI:0.01, 0.58) but not in China (effect: 0.36, CI: −0.01, 0.73), PNG (effect: 0.23, CI: −0.29, 0.75) or Sri Lanka (effect: 0.71, CI: −0.52, 1.94).

### Multivariate model controlling for FCT, gender inequitable attitudes, father's age and education

**FIGURE 3:**
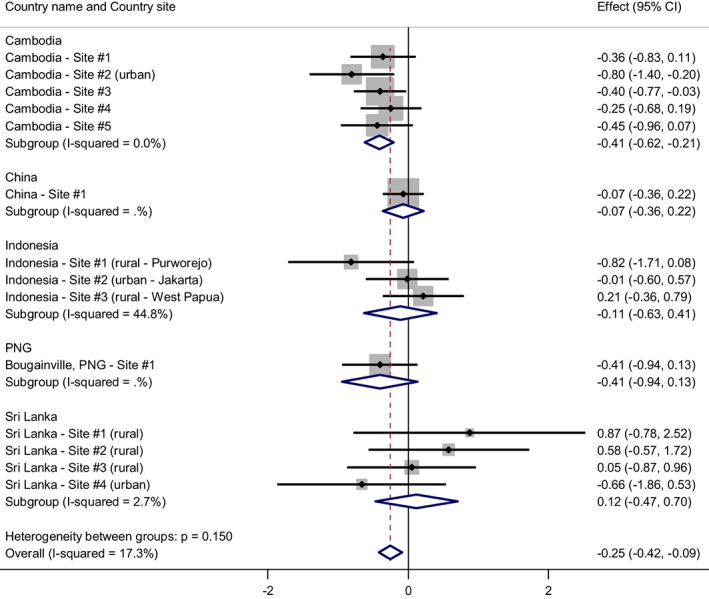
The relationship between heavy episodic drinking and fathering involvement, adjusting for father's experience of child trauma, father's gender equitable attitudes, father's education and father's age. *Note*: Weights are from random‐effects model

We analyzed the relationship between HED and fathering involvement, including gender equitable attitudes, FCT, father's age and low education in the model (results shown in Figure 3). The association remained significant in Cambodia and overall (*p* = 0.002), and this relationship varied only slightly (the effect size *increased* to −0.26) with the inclusion of fathers' gender‐equitable attitudes, experience of childhood trauma, father's age and education.

## DISCUSSION

This study aimed to analyze the relationship between HED and fathering involvement, consider the potential moderating effect of fathers' experiences of childhood trauma on this relationship, and control for possible confounding of HED with men's attitudes toward gender equality in five countries. Respondents overall reported significantly less fathering involvement if they reported HED, with this relationship significant in PNG and Cambodia, and in the same direction in Indonesia, but not evident in China and Sri Lanka. No significant interaction of HED with FCT was identified, and minimal confounding was present. After adjusting for gender equitable attitudes, FCT and father's education and age, the relationship between HED and fathering involvement remained significant overall and in Cambodia, with it no longer significant (CI: −1.00, 0.07) in PNG.

Previous research has found that HED is related to other aspects of men's lives, including their parenting (Kachadourian et al., 2009). Alcohol‐related harm from others' drinking to children has been identified across the world (Fulu et al., 2017; Laslett, Stanesby, et al., 2020). Some fathers are more likely to use physical discipline and physical forms of child abuse when they are unable to cope with difficult parenting situations and/or when they are intoxicated (Lee et al., 2011; Tamutienė & Laslett, 2016). In Uganda, Mehus et al. (2021) reported how drinking was associated with use of more severe punishment by parents. Men's heavy drinking often precedes and is associated with family violence (Cafferky et al., 2018; Jewkes et al., 2020; Leonard & Quigley, 2017; Wilson et al., 2017). Across cultures and time, research has found a consistent relationship between heavier drinking and IPV (Cafferky et al., 2018; Graham et al., 2011), including in previous analyses of the present data which found that drinking was associated with IPV after adjusting for gender inequitable attitudes (Laslett, Graham, et al., 2021). When children and mothers are affected by family violence, they may disengage from fathers. Reduced involvement could be related to less active involvement by either the father or children and their mothers. This paper provides new evidence that men in some countries are less likely to be involved with their children in positive ways if they drink heavily.

The results are important because data on the relationship of alcohol consumption with men's behaviors in families in these five countries (and other LMIC) countries are very rare. Thus, although the relationship between heavy drinking and less parental involvement was weak and not evident in all countries, it provides important directions for future research. This could include more in‐depth measures of parental involvement than was possible in this survey that covered many topics, including assessing different behaviors by age of the children. It would also be valuable to explore the relationship of drinking with intensity of both positive and negative parental involvement – that is, while parental involvement is seen as a positive father behavior, some parental involvement can be controlling and negative.

Notwithstanding the fact that some fathers involved in parenting may also be violent, our findings on heavy drinking and less involvement with parenting fit with a spectrum of evidence wherein less positive and more negative parenting, including child maltreatment, are associated with HED. More than 10 years ago, research identified paternal alcoholism, depression and marital satisfaction as important factors in parenting. Since then, alcohol and parenting literature has rarely focused on what it is about drinking in problematic ways that might make men less involved with their children (Kachadourian et al., 2009), particularly in lower‐income countries.

Contrary to our expectations, we did not find overall that FCT was directly associated with decreased involvement in parenting or that it moderated the effect of HED on fathering involvement.

We explored the relationship between fathering involvement and gender‐inequitable attitudes and a weak effect was found overall (*p* = 0.07). In some countries, specifically, in China and Sri Lanka, men with more gender equitable attitudes were significantly more likely to be involved in parenting. Given that the country‐level effect estimates were all positive, except in Cambodia where the result was marginally negative (−0.01), future research that includes a larger number of countries may find stronger evidence of a direct relationship.

We expected a possible interaction whereby gender‐inequitable attitudes increased the effect of HED on fathering involvement, we did not find this. Future research including alternative measures of fathering involvement that are more culturally applicable and/or more detailed and geared to age of children should be incorporated into future studies. However, our evidence did identify a relationship between gender equitable attitudes and greater fathering involvement in Sri Lanka and China. There was statistically significant evidence of this relationship, consistent with previous research (Bulanda, 2004; McGill, 2014) while there was no significant effect of HED. The present analyses pose questions for future research to better understand the culture aspects of men's alcohol consumption. These include: Why would gender equitable attitudes matter more in Sri Lanka and China yet not overall? And, why does HED matter more in Cambodia and more consistently across all countries but less so in Sri Lanka than elsewhere?

The overall evidence fits with understandings of gendered HED. Lesch and Adams (2016) reported on the gendered normativity of drinking in a low‐income farming community in South Africa. Their study described how societal norms supported men's drinking and male ways to drink. They reported how there were pressures on men to work long hours away from home and socialize with colleagues after work, often including drinking, with this sometimes including aggressive behaviors toward their families. Both decreased time with children and aggression likely hinder positive involvement with children.

As noted above, we found evidence of a significant association between gender inequitable attitudes and fathering involvement in China and Sri Lanka but not overall. When both gender attitudes and HED were included in the analysis there was only very limited (non‐significant) changes in the effect estimates for the relationship of fathering involvement with both HED and gender‐equitable attitudes. This somewhat contrasts with previous research with young people (not fathers), where masculine norms have explained patterns of problematic drinking among men (Iwamoto et al., 2011) and drinking consequences, both directly and indirectly via HED (Wells et al., 2014).

Our findings suggest tentatively that a reduction in HED may result in greater positive family involvement in families by men. Potentially, this could lead to direct effects upon women and children's health, safety, finances and wellbeing (Leonard & Quigley, 2017). There is plenty of evidence that alcohol causes a range of harms to families and children (Laslett, Jiang, et al., 2020; Laslett, Mojica‐Perez, et al., 2021; Laslett, Stanesby, et al., 2020) and this study highlights how reductions in drinking may be associated with more positive outcomes. Programs that reduce HED among men may well mean men have more time to spend with their children. Moreover, drinking per se may be an indicator of gender inequality and inequity, in which case gender transformative interventions (e.g., interventions that promote broader involvement by men in parenting and household/family roles) could strengthen father involvement in parenting if implemented, and a byproduct of this could be a secondary reduction in alcohol consumption. We have seen that gender transformative interventions such as Stepping Stones can do this (Gibbs et al., 2015). There is optimism that gender‐transformative approaches with men and boys can lead to better parenting and relationship skills with reduced levels of violence and better outcomes for families (Barker et al., 2010). Future studies should include both negative and positive measures of important outcomes for families and children, including family violence and parenting involvement.

### Limitations

This study relies on self‐report survey data, and it is likely that positive interviewer response bias may have been evident, leading to underestimates of poor fathering and other variables associated with stigma, including heavy drinking. Parenting is a multidimensional construct. The present study relies on a 3‐item measure of parental involvement that covers one dimension of positive parenting. The age of the child or children was not included in this analysis. While it would be useful to undertake analyses separating fathers into groups who had children of different ages, only the ages of the youngest and oldest children were asked about in the survey. Father's age was considered in this analysis and likely acts as a rough proxy for age of children, suggesting here that older fathers were more likely to report greater involvement in their [older] children's lives. In this study, we used a relatively limited definition of gender‐equitable attitudes, the GEM scale, and it is a scale that might be considered less related to measuring attitudes consistent with involvement in fathering. Although the GEM attitudes scale has been widely used, other measures, for example, the Conformity to Masculine Norms Inventory (CMNI) may produce more robust measures of masculinity. The CMNI is multi‐dimensional and has been found to be positively and negatively associated with various alcohol outcomes (Mahalik et al., 2003) and should be considered in future research.

The AUDIT (Saunders et al., 1993) was used to estimate HED using two of its standard questions on quantity consumed per occasion. Direct comparisons of alcohol consumption across countries is difficult because of difficulties in estimating “standard drinks”; however, these differences are unlikely to impact the relationship between HED and parental involvement given analyses by region, country and overall. A possible limitation is the use of a categorical measure of heavy drinking (HED vs. non‐HED). We believe that this dichotomy is the best approach given the large number of abstainers in some countries and a drinking pattern of infrequent drinking but heavy drinking when drinking occurs by men in some of these countries. However, we conducted a supplementary analysis using data from drinkers (excluding abstainers) to further explore the relationship between drinking and parental involvement. This analysis showed a similar effect: among drinkers, a higher number of drinks consumed on a typical occasion was significantly associated with less parental involvement with children overall (*n* = 1517, reg coefficient: −0.06, *p* value: 0.011) and in Cambodia. These results are presented in Table S1.

Stigma associated with reporting of heavy consumption of alcohol, particularly within cultures where drinking is disapproved of, may have led to underreporting of consumption, as described elsewhere and can be up to 40 or 50% (Livingston & Callinan, 2015). The hypotheses were not pre‐registered as this study utilized secondary data from the UNMCS. The study samples were randomly selected from within regions. However, these regions were purposively selected or self‐weighted, so should not be considered representative of entire countries in the study. Inclusion of more countries in the study would provide a greater opportunity to gain a broader overall assessment.

A further limitation is that our study did not examine cultural values (saving face, social norms, filial piety), worldviews (collectivism vs. individualism) and expectations within these countries. These are important factors within Asian countries and should be considered for inclusion in future UNMCS surveys, particularly given specific cultural values may be protective and linked to fathers' involvement with their children.

Despite these limitations and given cross‐cultural detailed studies of parenting involvement are rare, especially for the countries included in these analyses, this study provided a unique opportunity to explore drinking and fathering involvement. Overall, the primary effects were relatively small and future studies using larger surveys, in depth interviews and ethnographic studies would add further explanations of country‐level differences.

## CONCLUSIONS

This study provides evidence that fathers' HED is associated with less involvement in parenting. These findings suggest that reducing men's HED (and increasing gender‐equitable attitudes in some countries) may increase men's positive involvement in parenting.

## FUNDING INFORMATION

Laslett was funded by the Australian Research Council Grant DE190100329 and veski. The Victorian Near‐miss Award Pilot is being administered by veski for the Victorian Health and Medical Research Workforce Project on behalf of the Victorian Government and the Association of Australian Medical Research Institutes. Funding for the Pilot has been provided by the Victorian Department of Jobs, Precincts and Regions. The Victorian Near‐miss Awards are provided to eligible individuals who narrowly missed out on the 2021 NHMRC Investigator Grant funding in the Emerging Leaders 2 stream.

## CONFLICT OF INTEREST

None.

## Supporting information


Appendix S1
