# Supplementary material for: The relationship between fathers' heavy episodic drinking and fathering involvement in five Asia‐Pacific countries: An individual participant data meta‐analysis
Source: Alcohol Clin Exp Res. 2022 Dec 16;46(12):2137–48. doi: 10.1111/acer.14955 (PMC10108151; doi:10.1111/acer.14955)
Supplement: Supplementary file 1 — Appendix S1 [file ACER-46-2137-s001.docx]

**Supplementary analysis of the interaction between fathers’ childhood trauma and heavy episodic drinking on fathering involvement**

Figure S1a presents the univariate relationship between fathers’ childhood trauma and fathering involvement.

**Figure S1a The association between fathers’ childhood trauma and fathering involvement**

We hypothesised that there would be a moderation effect of father’s childhood trauma on the relationship between HED and fathering involvement. Figure S1b illustrates that there was no moderation evident overall but there was evidence of moderation in one country (PNG).

**Figure S1b The interaction between fathers’ childhood trauma and heavy episodic drinking on fathering involvement**

**Supplementary analysis of father’s drinking (number of usual drinks) and engagement with children**

One limitation of this study is that HED drinking variable we use (vs non-HED or abstaining), derived from a limited version of the AUDIT, includes respondents who report drinking 6 drinks or more on the one occasion. The use of this more inclusive and arbitrary definition of HED was indicated because we were using secondary data and because drinking is less common in some of the countries studied. A supplementary analysis using data from drinkers (excluding abstainers), showed that among those who do drink, an increasing number of drinks consumed on a typical occasion was associated with decreasing involvement with children overall (n=1,517, reg coefficient: -0.06, p value: 0.011) and in Cambodia. These results are presented in supplementary Table S1.

Table S1 Father’s drinking (number of usual drinks) and engagement with children

| Regression model: outcome father’s engagement score | Cambodia | China | Indonesia | PNG | Sri Lanka | Total^#^ |
| --- | --- | --- | --- | --- | --- | --- |
| N | 767 | 275 | 180 | 154 | 141 | 1,517 |
| **Co-efficient** | -0.12 | -0.07 | -0.01 | -0.13 | -0.18 | -0.06 |
| **p-value** | 0.001 | 0.317 | 0.864 | 0.052 | 0.080 | 0.011 |

**Supplementary analysis of the relationship between heavy episodic drinking and fathering involvement, excluding fathers whose oldest child is five years of age or lower**

We have undertaken a sensitivity analysis of the relationship between HED and fathering involvement that excludes fathers whose oldest children are aged 5 years or under in a sensitivity analysis. The results from analysing the reduced sample of 2,334 participants are essentially the same: with the overall effect in the entire sample being -0.23, (CI: -0.38, -0.09) c.f. with -0.24 (see Figure here below). We have noted this in the limitations and are happy to formally include this as an additional sensitivity analysis if requested. See Figure S2.

 …
**Figure R2/S2 The relationship between heavy episodic drinking and fathering involvement, excluding fathers whose oldest child is five years of age or lower.**
